# Government Debt Crisis and the Impact on National Health Systems: A Retrospective Study and Policy Recommendations to Greece

**DOI:** 10.7759/cureus.10786

**Published:** 2020-10-04

**Authors:** Evangelos Diamantis, Vasileios Charalampopoulos, Christos Damaskos, Paraskevi Farmaki, Nikolaos Garmpis, Anna Garmpi, Alexandros Patsouras, Georgios Kyriakos, Spyridon Savvanis, Vasiliki E Georgakopoulou, Nikolaos Trakas, Kostas Kounetas

**Affiliations:** 1 Internal Medicine: Diabetes and Endocrinology, Evangelismos Hospital, Athens, GRC; 2 Surgery, Laiko General Hospital, Medical School, National and Kapodistrian University of Athens, Athens, GRC; 3 Surgery, Renal Transplantation Unit, Laiko General Hospital, Athens, GRC; 4 Surgery, N.S. Christeas Laboratory of Experimental Surgery and Surgical Research, Medical School, National and Kapodistrian University of Athens, Athens, GRC; 5 Pediatrics, Agia Sofia Children’s Hospital, Medical School, National and Kapodistrian University of Athens, Athens, GRC; 6 Internal Medicine, Laiko General Hospital, Medical School, National and Kapodistrian University of Athens, Athens, GRC; 7 Internal Medicine, Tzaneio General Hospital, Pireas, GRC; 8 Internal Medicine: Diabetes and Endocrinology, Hospital General Universitario Santa Lucia, Cartagena, ESP; 9 Internal Medicine, General Hospital of Athens “Elpis”, Athens, GRC; 10 Internal Medicine: Pulmonology, Laiko General Hospital, Athens, GRC; 11 Internal Medicine: Pulmonology, Sismanogleio Hospital, Athens, GRC; 12 Laboratory Medicine, Sismanogleio Hospital, Athens, GRC; 13 Economics, University of Patras, Patras, GRC

**Keywords:** health care system, crisis, health quality

## Abstract

This article aims to explore the impact of the government debt crisis on the national health system (NHS) using a representative sample of respondents in Greek hospitals and provides certain suggestions regarding health policies that could be implemented at the national or local level. This study was conducted at the Evangelismos & Eye Polyclinic of Athens General Hospital in Athens, Greece. The study period was January and February of 2016, and the study included 600 outpatients who frequently submitted to follow-ups and consented to participate. Based on the results of this study, the participants had an average health status, while 94.2% of them had medical insurance. The predominant reason (88%) for choosing public hospitals instead of private practices was insufficient income. Further investigation revealed a significant positive correlation between the participant’s age and the number of hospital visits, the number of medical tests performed, and their satisfaction from the health services provided. Finally, a probit-model was used in order to study factors that could potentially influence their level of satisfaction from the services they used.

## Introduction

The 1976 charter of the World Health Organization (WHO) has defined health as “a state of complete physical, mental and social well-being” and not simply as the absence of disease or infirmity [1]. Thus, the concept of health is not only defined from a medical perspective, but also as something influenced by other factors, such as the environment, economy and finances, and employment. For instance, there is no doubt that a financial crisis along with a direct reduction of public health expenditure leads to unemployment and population impoverishment, eventually negatively affecting health indicators such as life expectancy, morbidity, mortality, access to health services, and weakening of the healthcare system [2-4].

In recent years, the global financial crisis, and the economic recession that followed, has imposed a heavy economic burden on citizens and the healthcare systems of the countries that suffer under the vicious circle created by the increase of unemployment, the loss of health insurance, decreases in available income, the inability of the people to bear the healthcare costs, and ultimately poor health outcomes resulting in increased morbidity and mortality through continuous impoverishment [5]. Greece, Spain, and Portugal have adopted strict fiscal austerity measures. As their economies continue to struggle, pressure on their national health systems (NHS) rises. In these countries, suicides and outbreaks of infectious diseases are becoming more common as health expenditure cuts limit the public's access to healthcare services [6]. The direct and indirect effects of the European financial crisis on the healthcare sector has been a debatable subject during the last years [5-8]. For instance, Rodriguez et al. studied any possible impact of the economic crisis in the health sector of the EU countries most affected by the crisis: Ireland, Greece, Spain, Italy, Cyprus, Latvia, Portugal, and Slovenia [9]. Among other things, the researchers concluded that the implementation measures resulted in a significant shift of the respective cost to households, either through an increase of co-payments for the use of health services or through higher fees for pharmaceutical products [9].

There is no doubt that the 2009 global economic crisis has had a significant impact on all sectors of the Greek economy and particularly on the NHS, which was already facing a number of problems and chronic issues [6]. In order to compensate for the budget cuts in healthcare, the Greek government has implemented a contribution system where the patients have to pay out-of-pocket money in addition to the provisions of their health insurance. However, with no signs of economic recovery despite eight years of austerity policies, the present and future status of the Greek NHS looks uncertain. A systematic review of the existing literature published from January to March 2009 on the impact of the economic crisis on public health and healthcare in Greece revealed that recent efforts to reform the Greek NHS focused mainly on short-term effects, such as expenditure reduction, while the necessary measures appear to have dubious long-term consequences for the Greek public healthcare system [8].

The aim of this study is to evaluate the impact of the government debt crisis on the NHS using a representative sample of Greek hospitals and addressing the question of whether the financial crisis has affected the use of NHS and health insurance by the citizens. Additionally, it provides suggestions regarding appropriate policies that should be implemented in order to avoid further deterioration in the sphere of public health.

## Materials and methods

Firstly, we statistically analyzed the answers of the participants using descriptive statistics. Then, we attempted to provide an initial estimation of the possible reasons that may influence the degree of patients’ satisfaction. We assumed that each patient could achieve a maximum level of satisfaction, by selecting treatment, which can be considered as a linear function, of the formula:

 s_i_* = β'x+e_i_ (1)

Where x is a vector of k x 1 exogenous variables, β is a vector of parameters to estimate and e_i__ _is a disturbance term, normally distributed with zero mean and constant variance. The variable s_i_* is unobservable. On the other hand, it is observed that values of the variable s_i_ corresponding to values of the variable s_i_* are:

s0 = 0 if s_i_* ≤ 0 (not satisfied)

s1 = 1 if 1 < s_i_* (satisfied) (2)

The relation (2) in fact illustrates a filter mechanism (censoring) while in the specimen is introduced a selectivity bias, which can be corrected by adding a selection mechanism as follows [10]:

θ*= a’z+u

*θ*=1 if θ* >0

*θ*=0 if θ* ≤0 (3a)

and

Pr(*θ*=1)=Φ(a’z)

Pr(*θ*=0)=1-Φ(a’z) (3b)

where θ* is the non-satisfaction level, z is a set of exogenous variables, Φ(.) is the cumulative distribution function for a standard normal distribution and u ~ N (0,1). If we consider as* θ*=1 the fact that the patient receives full satisfaction and 0 otherwise, the model as described by equations (3a) and (3b) is a multivariate probit model and s_k _is observed if and only if *θ*=1. Equation (1) can be defined as a sample selection problem (Heckman, 1979):

*E* [S│x, θ*>0]=β’χ +*E *[e│x, θ*>0] (4)

If e and u follow a bivariate distribution with the correlation coefficient, then the equation (4) becomes:

*E* [S│x, θ*>0]=*E *[e│x,u > -a’z]= *ρλ* (5)

where λ is defined as the ratio of the probability density function and the cumulative distribution function for standard normal variable:

λ=𝜑(−a′𝑧) / Φ(a'z) (6)

The estimation of equations (3a) and (3b) can be done by the maximum likelihood method. Consistent and effective asymptotic estimators of the model parameters are obtained by maximizing the likelihood function [10,11]. We find the marginal effects of the explanatory variables on the probability as

θ Prob (s=0) / θx= -φ (β’x+*ρλ*) β

θ Prob (s=1) / θx=[φ(-β'x-*ρλ*)-(μ-β'x-*ρλ*) β (7)

A large sample of 600 Greek public hospital users was recruited for the study. A survey was conducted from January 11, 2016, to February 11, 2016, at the Evangelismos & Eye Polyclinic of Athens General Hospital. Based on the data provided by the outpatients’ department administration, 75 people visit the hospital on a daily basis for examination by physicians on average. In order to draw firm conclusions from a representative sample, 600 questionnaires were distributed and completed, representing 40% of the calculated number of patients visiting the hospital within this period. This number of participants allowed us a 95% confidence level with a confidence interval of 3%.

## Results

Current state of knowledge

Out of the 600 participants, 35.3% were males and the remaining 64.7% were females. The majority of patients were over 60 years of age (35.3%), followed by those who were 50-59 years old (20.5%). The majority of the participants were married (49.2%). Regarding the level of education, 36.8% of patients had completed higher education, followed by 29.7% who were high school/post-high school graduates [institute of professional training (IPT)]. The majority of patients reported that they were pensioners (32.2%), followed by private-sector employees (20.7%), and civil servants (15.7%). Finally, most participants (47.2%) stated that their monthly income was less than or equal to €600, followed by 20.2% who reported an income between €1000-1500, and 18.2% who stated an income between €600-800 (Table 1).

In the second part of the survey, respondents were asked to answer questions related to their health status. Regarding the general state of their health, 35.3% of patients replied that their health was in a moderate state, followed by those who reported a poor (28.5%) or very poor health (18.5%). They were also asked if they followed a specific pharmaceutical treatment on a regular basis, and 68.5% of them responded positively. Approximately three out of four patients (75.7%) also responded positively about receiving a specific type of treatment. Most of the participants (67.2%) stated that they had a chronic problem and had to visit the hospital regularly. The vast majority (94.2%) had health insurance (Table 2).

The third part of the survey included questions regarding the visits to the hospital. The frequency of visits to a public hospital was one to five times per year for 59.2% of patients, followed by 26.3% who visited one to five times a semester. Two-thirds (73.8%) of patients had visited public hospitals even before 2009 (Table 3). The most important reason why the participants did not visit private hospitals was their low income (88%) (Table 4).

In the third section of the questionnaire, participants were asked about whether they felt that the crisis had affected their visits rate to private doctors, visits to private clinics, and visits to public hospitals. As shown in Table 5, most participants (41%) stated that they visited private doctors less frequently, similarly to less frequent visits to private clinics (45%). Visits to public hospitals also decreased, but at a slightly lower rate (36%).

The fourth part of the survey included questions on knowledge and patients' perception of out-of-pocket share and private expenditure. Initially, participants were asked to answer about whether they knew that they had to pay an amount of money as a contribution whenever visiting public doctors to examine them, to get medical prescriptions, or to undergo laboratory tests. Most of them (76.5%) said that they were aware of this contribution system (Table 6). Then the participants answered about how often they acquired their medication in the last 12 months. Most of the patients (86.8%) acquired most of their prescribed medications and used it as instructed by the physician (Table 7). Other patients did not acquire their medication either occasionally (5%) or permanently (0.7%) due to financial constraints.

Then followed a series of questions regarding the patients’ opinion on how they had been affected by the implementation of the contribution system and the increase of the contribution percentage. As shown in Table 8, most agreed that the implementation of the contribution system and the increase of the contribution percentage had rather reduced unnecessary visits to the outpatient clinic of the hospitals by 41.5%; improper use, unnecessary discharge, and abuse of medication by 32.7%; and non-diagnostic and unnecessary laboratory tests by 32.2%. Approximately, one-third of the patients (38.8%) also agreed that the implementation of this system had directed a small number of people to the private sector. According to them, this system had made people think twice before visiting the doctor (34.3%) and had slightly reduced the compliance of people with their suggested treatment (33.3%). Finally, many of the participants (42.3%) agreed that the money saved by implementing these measures would definitely not be used to improve health services.

**Figure 1:**
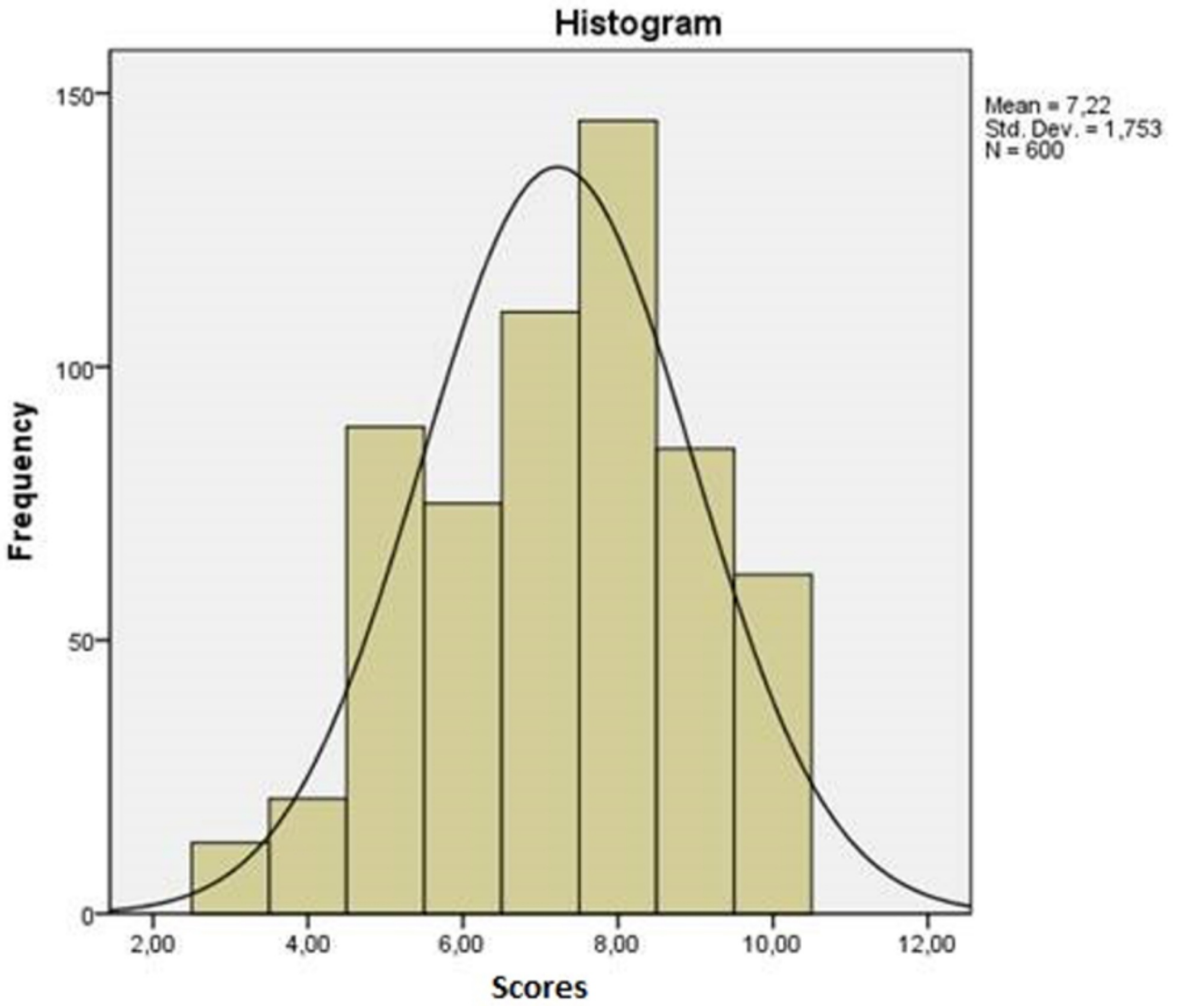
Frequency histogram showing the ratings given by the participants on their overall experience of the public hospital (0=very bad, 10=excellent)

The fifth and final part of the research was to investigate the satisfaction of the participants with the offered health services. Initially, they were asked how satisfied they were with the services provided by public hospitals; 35.7% responded that they were satisfied and 28.2% said they were very satisfied (Table 9). Then the participants were asked to rate the provided healthcare, taking into account their overall experience in the public hospital. It should be noted that most ratings were higher than 5 and the average overall score was 7.22 (Table 10, Figure 1).

In the last section, participants were asked to suggest as to what subject they would like to be more informed on. Most participants asked for more information on their rights as patients and the use of health services (40.8%) and information regarding their health insurance or the National Provider of Health Services of Greece, founded in 2011 (31.7%) (Table 11).

Further analysis was performed in order to identify possible correlations between variables. Initially, a correlation between age and visit frequency to a state hospital was performed. The x2 control found that these two variables had a positive association (p = 0.000) and Pearson control found a negative association (r = -0,298). This means that the older the participants, the more frequent their visits to a hospital.

Next, we analyzed how age affected the frequency of patients undergoing laboratory tests. By the non-parametric Kruskal Wallis control, it was found that these variables were positively associated (p = 0.037) and Pearson control found a slightly positive correlation (r = 0.008). This means that the older the participant, the more frequent a laboratory test is undertaken by the participant.

Finally, a correlation between age and the total score for the offered health services was conducted. The x2 control found that these two variables were positively associated (p = 0.000) and Pearson control found a slightly positive correlation (r = 0,097). This means that the older the participant, the greater the satisfaction of the participant.

The main aim of our study was to identify the factors that determined the level of patients’ satisfaction based on the received information. As already evident from the theoretical framework and, in order to reach the aim of the study, our empirical approach had to be adjusted, depending on the relations (3b) and (7), as portrayed above.

In our approach, we used variables drawn from the questionnaire distributed to patients of the hospital Evangelismos & Eye Polyclinic of Athens General Hospital. Initially, we looked for an important set of explanatory variables among the available basic economic variables as described in previously published related literature, as well as the transformation or the interactions between those variables. These variables included characteristics of the patients who participated in the field research concerning the level of education and income, marital status, frequency of treatment, and certain characteristics regarding their treatment. Finally, we used variables that correlated the level of satisfaction of patients with the provided healthcare they received. Table 12 lists the variables and their respective statistical measures.

Secondly, we wanted to use models with the best econometric properties among other alternatives, which could be calculated from the available data and the set of available variables. This implies that the variables that do not show statistically significant results have been included in our final model since this is also an important finding. The initial hypothesis was that the coefficients of the explanatory variables were statistically different from zero, and a joint test-to-test hypothesis was that all parameters associated with the explanatory variables were statistically different from zero. A series of separate tests related to the control of our initial hypothesis was conducted. A model adaptation measure to the data (goodness-of-fit measure) commonly referred to as McFadden's measure, as rho-square [10], was evaluated, as well as the percentage of correctly predicted values. Furthermore, the specificity test of the chosen model included a homoscedasticity test [11], and control for the omission of certain variables. The omission of a statistically significant variable in the context of a binary, dichotomous choice model implies that even if the omitted variable is uncorrelated with the included, the coefficient of this variable will be inconsistent [12].

Determinants of satisfaction level

The research question was examined using a probit type model, which describes the level of satisfaction that patients receive from the hospital Evangelismos & Eye Polyclinic of Athens General Hospital, as reflected by the dependent binary variable. At this point, we should mention that according to our questionnaire, the specific variable took five successive values for assessment purposes but was converted into binaries where 1 corresponded to significant/very much, while the value 0 corresponded to insignificant/very little. This model was also used to "predict" whether a randomly patient hospitalized in this hospital would be satisfied or not. It was also used to examine what factors influenced this probability.

The maximum probability estimates of the independent variables and basic statistics of the probit model are shown on the left side of Table 13. The value of the X2 test rejects the hypothesis that the combined effect of all explanatory variables is statistically equal to zero. The model used predicted correctly at 89.02% (528 of 600) rate of observations. In the same table, the marginal effects only for the statistically significant variables are given, since the concept of marginal effects is meaningless for the variables that are statistically insignificant. Ten of the 13 variables included in our model were statistically significant at the 5% significance level, while another variable was at a 10% significance level.

It was found that having health insurance was insignificant, as was the variable that reflected the fact that the responding patient graduated from a university or a technological institute (as a sign of a higher level of education). Based on the results of our model, we can conclude that the variables that capture the patients’ level of income have a statistically significant effect on the probability of the particular patient believing that the level of satisfaction is quite high. Indeed, this probability is affected by variables reflecting the external difficulties faced by patients and connects the level of income that they hold in a specific period of time with their desire for better services.

The variable that reflected the fact that the respondent patient was unemployed was also significant and had also a negative effect. From the variables that were statistically significant but had a negative effect, we should note the variable of chronic health problems. This variable reflected a longer-term relationship between the disease level of the respondents and the level of service received. Finally, the effect of the variable that reflected the fact of the treatment plane (long-short-term) with the satisfaction level followed the same trend.

On the other hand, according to the empirical results, the variable that demonstrated the education level seemed to have a mixed character. Patients with basic education assessed as positive and statistically significant the possibility that the health services would be characterized as significantly good to very good. On the other hand, patients with high school level education evaluated the same service negatively. Finally, the influence of gender was statistically significant and had a positive correlation, with men positively evaluating the level of satisfaction. Table 13 shows the limit effects that reflect the change there will be in the satisfaction probability from the change of one unit of the explanatory variables ceteris paribus.

At this point, it should be noted that various other variables included as explanatory variables were not only statistically significant, but their use also led to a decrease in the percentage of correctly predicted cases. These variables included the possession of health insurance (VIVL) and a higher level of education (EDUCL3).

**Table 1:** Demographics of the participants IPT: institute of professional training; TI: technological institute

Gender	Νumber of patients (n)	Percentage
Male	212	35.3
Female	388	64.7
Αge (years)		
≤20	9	1.5
20-29	93	15.5
30-39	55	9.2
40-49	108	18
50-59	123	20.5
>60	212	35.5
Μarital status		
Μarried	295	49.2
Single	138	23
Widowed	69	11.5
Divorced	98	16.3
Εducation level		
Primary	74	12.3
Junior high	75	12.5
Ηigh/IPT	178	29.7
University/TI	221	36.8
Postgraduate	34	5.7
Doctorate	18	3
Ιncome (€)		
≤600	283	47.2
601-800	109	18.2
801-1000	70	11.7
1001-1500	121	20.2
>1500	17	2.8
Employment type		
Public servant	94	15.7
Private employee	124	20.7
Self-employed	45	7.5
Pensioner	193	32.2
Ηousekeeping	47	7.8
Student/soldier	18	3
Unemployed	79	13.2

**Table 2:** Health status of the participants

Overall health status	Number of patients	Percentage
	(n)	(%)
Very good	58	9.7
Good	48	8
Moderate	212	35.3
Bad	171	28.5
Very bad	111	18.5
Medication		
Yes	411	68.5
No	189	31.5
Therapy		
Yes	454	75.7
No	146	24.3
Chronic problem		
Yes	403	67.2
No	197	32.8
Medical booklet		
Yes	565	94.2
No	35	5.8

**Table 3:** Visits of the participants to state hospitals

Frequency of visits	Number of patients	Percentage
	(n)	(%)
1-5 times per week	25	4.2
1-5 times per month	62	10.3
1-5 times per semester	158	26.3
1-5 times per year	355	59.2
Visit before 2009		
Yes	443	73.8
No	157	26.2

**Table 4:** Reasons for which the participants do not visit public hospitals

Reasons for not visiting	Number of patients	Percentage
	(n)	(%)
Financial	528	88
Good medical staff	60	10
Prompt service	1	0.2
Lack of trust in some private medical centers	11	1.8

**Table 5:** Degree to which the crisis has affected visits to private doctors, visits to private clinics/labs, and visits to public hospitals

To what degree has the crisis affected:	Number of patients	Percentage
	(n)	(%)
Rate of visits to private doctors		
None	49	8.2
Quite	136	22.7
Significantly	169	28.2
Very much	246	41
Visits to private clinics/labs		
None	39	6.5
Slightly	7	1.2
Quite	109	18.2
Significantly	175	29.2
Very much	270	45
Visits to public hospitals		
None	79	13.2
Slightly	59	9.8
Quite	95	15.8
Significantly	151	25.2
Very much	216	36

**Table 6:** Awareness of patients about out-of-pocket share and expenditure

Do you know that you have to pay a certain amount as a contribution every time you visit state doctors in order to examine you, prescribe medication, or order lab tests?	Number of patients	Percentage
	(n)	(%)
Yes	459	76.5
No	141	23.5

**Table 7:** Frequency of medication intake in the last 12 months

Medication intake	Number of patients	Percentage
	(n)	(%)
I bought it almost every time and I used it according to the doctor’s prescription	521	86.8
I bought it almost every time but I did not consider it necessary to use it all times	20	3.3
I bought it but sometimes I saved on doses	16	2.7
I did not buy it all times because I did not consider it necessary	9	1.5
Sometimes I did not buy it because I did not have money	30	5
I stopped buying it because I did not have money	4	0.7

**Table 8:** Consequences of the implementation of payments measure or increase in the participation fee

Characteristic	None	Slightly	Quite	Significantly	Very much
Has reduced unnecessary visits to the outpatient clinic of the hospital	142	125	249	37	47
	-23,70%	-20,80%	-41,50%	-6.20%	-7.80%
Has reduced waste and abuse of medication	67	169	196	98	70
	-11.20%	-28.20%	-32.70%	-16.30%	-11.70%
Has reduced pointless and unnecessary lab tests	115	126	193	123	43
	-19.20%	-21%	-32.20%	-20.50%	-7.20%
The money saved will be used for the improvement of health services	254	178	121	42	5
	-42.30%	-29.70%	-20.20%	-7%	-0.80%
Has made some people go to the private sector	173	233	125	35	34
	-28.80%	-38.80%	-20.80%	-5.80%	-5.70%
Has made people think twice before going to the doctor	55	206	152	78	109
	-9.20%	-34.30%	-25.30%	-13%	-18.20%
Has reduced people’s compliance in terms of taking their treatment (e.g., they take a smaller dose or do not buy medication)	129	200	142	70	59
	-21.50%	-33.30%	-23.70%	-11.70%	-9.80%

**Table 9:** Satisfaction with the offered health services

Level of satisfaction	Number of patients	Percentage
	(n)	(%)
None	3	0.5
Slight	115	19.2
Quite	214	35.7
Significant	169	28.2
Very much	99	16.5

**Table 10:** Overall satisfaction with the offered health services

Score	Number of patients	Percentage
	(n)	(%)
3	13	2.2
4	21	3.5
5	89	14.8
6	75	12.5
7	110	18.3
8	145	24.2
9	85	14.2
10	62	10.3

**Table 11:** Subjects on which the participants would like to receive more information EOPYY: Greek acronym for the National Organization for the Provision of Health Services

Subject	Number of patients	Percentage
	(n)	(%)
Information about my health insurance (EOPYY)	190	31.7
Information about my rights regarding the use of health services	245	40.8
Information about access to hospitals	27	4.5
Information about access to primary healthcare services	90	15
Information about access to general doctor or pathologist	28	4.7
Information about the cost of the visit to a doctor or health service	20	3.3

**Table 12:** Variables used in the econometric estimation

Variable	Mean	Standard deviation	Minimum	Maximum	Variable description
Satisfaction	0.446	0.497	0	1	Satisfaction level (1 if the patient is fully satisfied and 0 otherwise)
Sex	0.34	0.474	0	1	Gender (1 if male and 0 otherwise)
Spoudes1	0.435	1.169	0	7	Education level 1 (1 if only mandatory education and 0 otherwise)
Spoudes2	0.085	0.279	0	1	Education level 2 (1 if high school graduate and 0 otherwise)
Spoudes3	0.127	0.247	0	1	Education level 3 (1 if university/technological Institute graduate and 0 otherwise)
Therapy	0.756	0.429	0	1	1 if the patient undertakes a treatment and 0 otherwise
Medication	0.673	0.469	0	1	1 if the patient regularly takes medication and 0 otherwise
Provlimayg	0.671	0.469	0	1	1 if the patient has a chronic health problem and 0 otherwise
Vivliario	0.941	0.234	0	1	Health booklet (1 if exists and 0 otherwise)
Occup1	0.131	0.338	0	1	Working status (1 if employed and 0 otherwise)
Inc1	0.471	0.499	0	1	Income 1 (1 if the wage was less than or equal to €600)
Inc2	0.181	0.385	0	1	Income 2 (1 if the wage was €600-800)
Inc3	0.116	0.321	0	1	Income 3 (1 if the wage was €800-1000)
inc4	0.201	0.401	0	1	Income 4 (1 if the wage €1000-1500)

**Table 13:** Estimated variables and marginal effects of the basic model: dependent variable Rate of correct predictions in total observations = 79.42%; numbers in parenthesis represent asymptomatic ratio t *Statistical significance of 5%; **Statistical significance of 10%

Variables	Estimated factor	Marginal Effects		Variables		Estimated factor	Marginal Effects	
CONSTANT	0.207 (2.32)	0.189 (3.42)		SEX		-1.024 (-4.51)*	-0.391 (-4.59)	
EDUCL1	0.381 (3.48) *	0.15 (3.45)		VIVL		0.363 (1.37)	-	
EDUCL2	-0.728 (-2.76)*	-0.258 (0.001)		OCCUP1		-1.087 (-5.31)*	-0.361 (-7.17)	
EDUCL3	-0.169 (-1.18)	-		INC1		-1.27 (-2.91)*	-0.468 (-3.32)	
THERAP	-0.433 (-1.98)**	-0.171 (-1.98)		INC2		-2.224 (-4.98)*	-0.579 (-10.15)	
MEDICAT	1.197 (5.56)*	0.42 (6.63)		INC3		-1.356 (-2.91)*	-0.415 (-4.59)	
SEX	0.480 (3.42)*	0.035 (2.93)		INC4		-1.964 (-4.31)*	-0.555 (-7.74)	

## Discussion

The primary aim of this study was to investigate the effects of the economic crisis on the health system and the access of citizens to health services during the crisis. The secondary aim was to make certain suggestions concerning the health policies based on our findings. These suggestions could be implemented at the national and local levels. We also attempted to answer a number of other questions: what percentage of citizens did not have any health insurance? Does financial strain stop citizens from visiting a hospital? Is the frequency of hospital visits affected by age? Is the number of performed laboratory tests affected by age? Is the level of satisfaction with the services provided affected by age?

Initially, the study recruited 600 people, the majority of whom were women. The majority of the sample was over the age of 60 years and was married. Regarding the level of education, most of the participants were university/technological institute graduates and most were pensioners. Finally, most participants stated that their monthly income was less than or equal to €600, followed by those who stated that their income was between €1000-1500.

In the second part of the survey, respondents were asked to answer questions related to their health status. Regarding the state of their health in general, most participants said that it was moderate. Also, the majority said that they regularly took specific medications and followed specific treatments. Most had a chronic health problem and needed to frequently visit a doctor/hospital. Finally, 94.2% of patients had health insurance.

The third part of the survey included questions about their visit to the hospital grounds. Most patients visited the hospital one to five times a year, with 73.8% stating that they had used to visit a hospital before 2009 as well. The most important reason for not visiting a public hospital was financial strain (88%). Most participants believed that there was a decrease in the number of visits to private doctors, private hospitals, and public hospitals since the beginning of the crisis.

The fourth part of the survey included questions on patients’ knowledge and perception of contribution fees and private expenditure. The results showed that most had good knowledge of both. Regarding the frequency of patients acquiring their medication, most of them bought their medication almost every time and used them according to the doctor’s prescription. This was followed by a series of questions about the opinion of the participants about how they had been affected by the implementation of the contribution system and the increase in the contribution percentage. Based on our results, most patients agreed that these were slightly limiting unnecessary visits to the outpatient clinic of the hospitals; these had also decreased the abuse and wastage of medication and decreased non-diagnostic and unnecessary laboratory tests. Furthermore, most also agreed that the implementation of these measures had directed a small number of patients to the private sector, but it had not made people think twice before visiting the doctor. It had also limited to a small degree the compliance of patients in terms of receiving their medication. Finally, the majority of participants agreed that the money saved from these measures would definitely not be used to improve health services.

The fifth and final part of the research entailed investigating the satisfaction of the participants with the offered health services. Most respondents were fairly or very satisfied, while the average score of the provided healthcare, taking into account their overall experience of the public hospital, was 7.22 out of 10. Finally, most participants stated that they wished to have more information with respect to their rights regarding the use of health services and about their health insurance or the National Provider of Health Services of Greece.

Finally, the correlations performed in order to detect dependencies between variables found that as age increased, the more frequent the visit of the participants to a hospital, the more common that the participants underwent laboratory tests, and the greater the satisfaction of participants.

The econometric estimation of the results showed that the variables associated with the patients’ incomes had a statistically significant negative effect on the probability of the particular patient to believe that the level of satisfaction was quite high. Unsurprisingly, the same effect showed the variables that reflected the fact that the respondent patient was unemployed as well as the ones that reflected a longer-term relationship between the disease level of the respondents and the level of service they received. Finally, the effect of the variable that pointed to the level of treatment (long-short) seemed to be on the same wavelength as the level of satisfaction.

In contrast, having health insurance and frequent patient visits to hospitals for treatment had a positive effect on patient’s satisfaction. Finally, the effect of the male gender was statistically significant and had a positive correlation with the level of satisfaction with the health services they received.

Interestingly, the study of the variable associated with the level of education seemed to have a mixed character. Specifically, patients with a basic level of education were largely satisfied with the level of health service unlike patients with high school education, who negatively evaluated this possibility.

## Conclusions

Based on our findings, there is an immediate need for a radical restructuring of the Greek healthcare system in order to provide high-quality services equitably, universally, and free at the point of delivery. This situation has come about due to the economic crisis of recent years. The reform process, so far, has been hasty and untargeted, and a number of strategies, procedures, and methods for the optimization of the National Health Service still need to be addressed by the government. As the data from our analysis indicate, the majority of people are dissatisfied with the quality of health provided and have decreased the frequency of their visits to doctors. Therefore, the Greek government should consider providing equitable access to services, which would lead to greater empowerment of citizens in decision-making about the services they need and their treatment options, and to the reconstruction of the health system towards a patient-centered, primary care system.
